# Supplementary material for: Sarcopenia and Mortality in Older Hemodialysis Patients
Source: Nutrients. 2022 Jun 5;14(11):2354. doi: 10.3390/nu14112354 (PMC9182960; doi:10.3390/nu14112354)
Supplement: Supplementary file 1 [file nutrients-14-02354-s001.zip › nutrients-1689347-supplementary.pdf]

Table S1. Characteristics of patients alive or deceased at 2 years according to sex. Data shown as mean ± SD, n (%).

	**Male**			**Female**		
	**Alive n=19 (46%)**	**Deceased n=22 (54%)**	**p value**	**Alive n=11 (58%)**	**Deceased n=8 (42%)**	**p value**
Demographic data						
Age (years)	78.7±4.5	83.6±5.8	**0.005**	81.9±5.1	84.5 (±5.4)	0.32
Dialysis vintage (months)	44.5±41.2	51.4±39.1	0,585	53.9±45.5	52.3 (±42)	0.558
Comorbidities						
Diabetes	9.0 (47.4%)	9.0 (40.9%)	0,78	9.0 (81.8%)	4.0 (50.0%)	0,141
Cardiovascular disease	11.0 (57.9%)	21.0 (95.5%)	**0,004**	4.0 (36.4%)	4.0 (50.0%)	0,552
Malignancy	6.0 (31.6%)	11.0 (50.0%)	0,233	3.0 (27.3%)	1.0 (12.5%)	0,435
Analytical data						
Albumin (g/dl)	3.7±0.4	3.7±0.4	0,476	3.7±0.4	3.4 ±0.8	0,284
Hemoglobin (g/dl)	11.5±1.1	11.3 ±1.0	0,682	11.6±0.9	10.8 ±1.3	0,119
C Reactive Protein (mg/L)	0.8±0.7	2.3 ±3.8	0,088	0.7±0.7	3.0 ±3.7	0,055
25OH Vitamin D_3 (ng/ml)	18.4±8.7	23.6 ±15.7	0,207	20.9±12.8	24.2 ±15.8	0,621
Kt/V_{urea}	1.7±0.4	1.7 ±0.4	0,546	2.1±0.3	1.9 ±0.3	0,061
Anthropometric data						
Body mass index (kg/m^2)	26.5±4.1	25.1 ±2.6	0.190	23.8±4.3	24.4 ±3.7	0.766
Mid-Upper Arm Circumference (cm)	27.5±2.8	25.9 ±2.3	0.065	25.4±3.5	26.2 ±2.6	0.621
Waist Perimeter (cm)	96.7±7.1	97.2 ±8.2	0.841	84.9±12.9	82.3 ±2.0	0.612
Hip Perimeter (cm)	102.3±7.3	101.0 ±6.7	0.557	96.4±8.7	101.5 ±4.2	0.178
Waist hip index	0.9±0.0	1.0 ±0.1	0.424	0.9±0.1	0.8 ±0.0	**0.028**
Tricipital Fold (mm)	1.2±0.5	1.0 ±0.3	0.296	1.3±0.5	1.6 ±0.4	0.300
Abdominal Fold (mm)	21.0 ±7.6	18.4 ±5.8	0.337	15.6 ±4.9	16.1±3.8	0.866
Subscapular Fold (mm)	17.0 ±6.8	15.9 ±6.7	0.641	13.5 ±9.5	12.8 ±3.8	0.865
Body composition						
Muscle Mass (kg)	22.8±2.8	19.4 ±2.7	**<0.001**	15.1±1.7	16.2 ±2.1	0.205
Fast Mass (kg)	23.7±6.1	22.2 ±4.1	0.342	21.5±8.8	22.7 ±5.5	0.741
Total Body Water (l)	37.7±4.4	33.6 ±5.1	**0.009**	24.8±3.1	26.9 ±3.0	0.158
Overhydration (l)	1.1±1.7	1.3 ±1.5	0.728	0.7±1.5	0.8 ±0.9	0.787

*p<0.05 in bold. **p Male vs Female

Table S2. Univariate hazard ratio (HR), 95% confidence interval (95% CI) for all-cause death by diagnostic algorithm.

Variable	**Value**	**n (%)**	**HR (95% CI)**
GSD	N	15 (27.3)	-
	Y	40 (72.7)	3.80 (0.51-28.11, p=0.411)
STS5	N	7 (11.7)	-
	Y	53 (88.3)	2.31 (0.55-9.72, p=0.252)
GSD+ASM	N	37 (61.7)	-
	Y	23 (38.3)	**3.00 (1.44-6.24, p=0.003)**
STS5+ASM	N	38 (63.3)	-
	Y	22 (36.7)	2.76 (1.34-5.71, p=0.006)
GSD &/or STS5+ ASM	N	36 (60.0)	-
	Y	24 (40.0)	2.68 (1.29-5.57, p=0.008)
GSD+ASM+GS	N	41 (68.3)	-
	Y	19 (31.7)	**3.33 (1.61-6.89, p=0.001)**
STS5+ASM+GS	N	41 (68.3)	-
	Y	19 (31.7)	**3.33 (1.61-6.89, p=0.001)**
GSD &/or STS5+ASM+GS	N	41 (68.3)	-
	Y	19 (31.7)	**3.33 (1.61-6.89, p=0.001)**
GSD+**ASM+TUG**	N	49 (81.7)	-
	Y	11 (18.3)	**5.60 (2.49-12.58, p<0.001)**
STS5+**ASM+TUG**	N	49 (81.7)	-
	Y	11 (18.3)	**5.60 (2.49-12.58, p<0.001)**
GDS &/or STS5+**ASM+TUG**	N	49 (81.7)	-
	Y	11 (18.3)	**5.60 (2.49-12.58, p<0.001)**
GSD+ASM+SPPB	N	40 (66.7)	-
	Y	20 (33.3)	**2.95 (1.43-6.09, p=0.003)**
STS5+ASM+SPPB	N	39 (65.0)	-
	Y	21 (35.0)	2.60 (1.26-5.37, p=0.009)
GDS &/or STS5+ASM+SPPB	N	39 (65.0)	-
	Y	21 (35.0)	2.60 (1.26-5.37, p=0.009)
GDS &/or STS5+ASM+GS &/or TUG &/or SPPB	N	38 (63.3)	-
	Y	22 (36.7)	2.76 (1.34-5.71, p=0.006)

N: no, Y: yes

Figure S1. Survival according to EGWSOP2 criteria at 24 months for non-COVID-19 mortality. COVID-19 deaths were censored

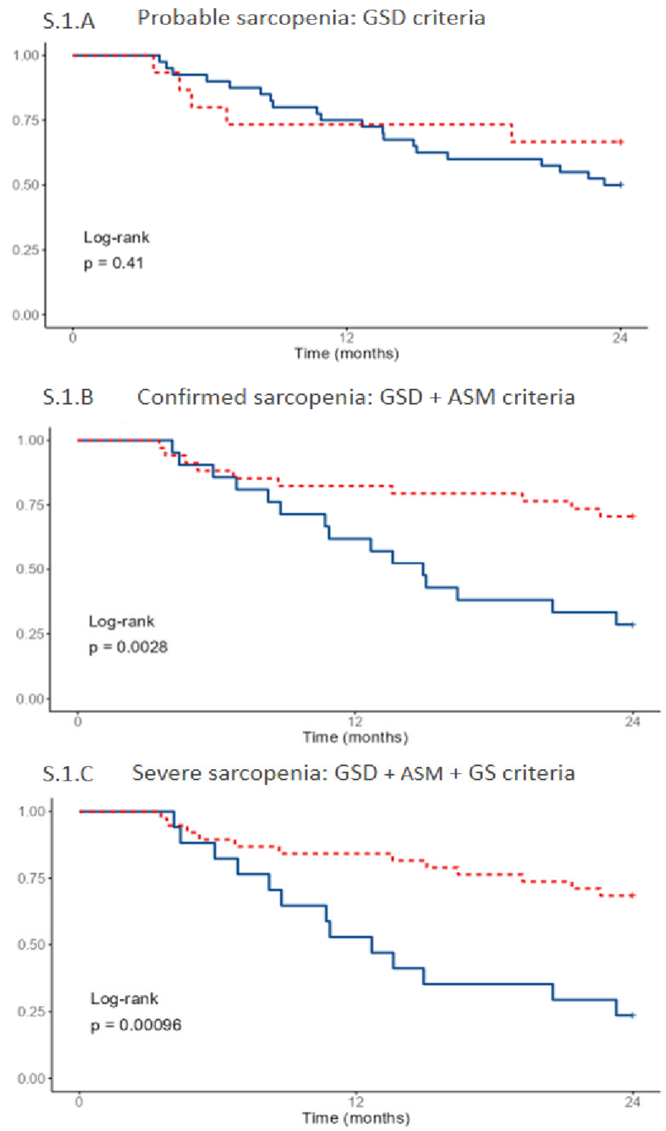

Vertical axis shows survival. Probable of severe sarcopenia in blue, no sarcopenia in discontinuous red.
Survivors, n=30. Deceased, n=25. GSD: grip strength by dynamometry,
ASM: appendicular skeletal muscle mass, GS: gait speed.

Figure S2. Non-COVID-19 multivariate mortality risk according to EGWSOP2 criteria at 24 months. Data shown as hazard ratio and 95% confidence interval.

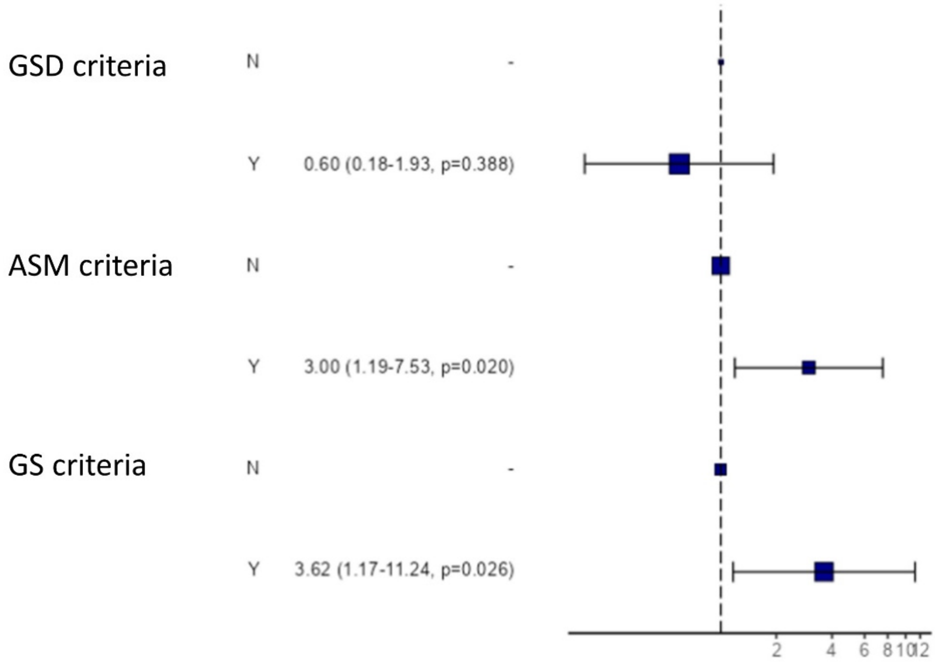

Survivors, n=30. Deceased, n=25. GSD: grip strength by dynamometry, ASM: appendicular skeletal muscle mass, GS: gait speed.
